# Approach to Thrombophilia in Pregnancy—A Narrative Review

**DOI:** 10.3390/medicina58050692

**Published:** 2022-05-23

**Authors:** Miruna Samfireag, Cristina Potre, Ovidiu Potre, Raluca Tudor, Teodora Hoinoiu, Andrei Anghel

**Affiliations:** 1 Department of Internal Medicine, Discipline of Clinical Practical Skills, “Victor Babes” University of Medicine and Pharmacy, No. 2 Eftimie Murgu Square, 300041 Timisoara, Romania; samfireag.miruna@umft.ro (M.S.); tstoichitoiu@umft.ro (T.H.); 2 Advanced Cardiology and Hemostaseology Research Center, “Victor Babes” University of Medicine and Pharmacy, No. 2 Eftimie Murgu Square, 300041 Timisoara, Romania; 3 Department of Internal Medicine, Discipline of Hematology, “Victor Babes” University of Medicine and Pharmacy, No. 2 Eftimie Murgu Square, 300041 Timisoara, Romania; potre.ovidiu@umft.ro; 4 Department of Neurosciences, Discipline of Neurology, “Victor Babes” University of Medicine and Pharmacy, No. 2 Eftimie Murgu Square, 300041 Timisoara, Romania; tudor.raluca@umft.ro; 5 Department of Biochemistry and Pharmacology, Discipline of Biochemistry, “Victor Babes” University of Medicine and Pharmacy, No. 2 Eftimie Murgu Square, 300041 Timisoara, Romania; biochim@umft.ro

**Keywords:** thrombophilia, pregnancy, genetic testing, screening, narrative review

## Abstract

Thrombophilia is a genetic predisposition to hypercoagulable states caused by acquired haemostasis conditions; pregnancy causes the haemostatic system to become hypercoagulable, which grows throughout the pregnancy and peaks around delivery. Genetic testing for thrombophilic gene mutations is evaluated using different methodologies of real-time polymerase chain reaction and DNA microarrays of specific genes. Adapting the general care of the pregnant woman to the particularities caused by thrombophilia is an important component, so screening is preferred to assess the degree of genetic damage that manifests itself as a risk of thrombosis. The major goal of this narrative review was to quantitatively evaluate the literature data on the specific care of pregnant women with thrombophilia that are at risk of developing unplanned miscarriages.

## 1. Introduction

Thrombophilia [1,2] is defined as a predisposition for thrombosis, occurred as a result to a genetic defect (hereditary); it may be diagnosed postnatally (acquired). Thrombosis develops secondary to the alteration of one or more components of the haemostasis, which includes coagulation factors, plasmatic proteins, blood flow, vascular surfaces and cellular elements that finally lead to a hypercoagulable state. This results in arterial or venous thrombosis. A reasonable approach of a patient with thrombosis starts with the attempt to characterize the hypercoagulable condition as hereditary or acquired. Pregnancy modifies the haemostatic system into a hypercoagulable condition, which is absolute around delivery. Recurrent pregnancy loss (RPL) is described as two or more unplanned miscarriages, which involves about 5% of women of reproductive age [3]. Novel studies indicate that thrombophilia is one of the causes that leads to RPL [2,3]. Hereditary states develop due to the presence of some mutations that change a gene that codifies a plasmatic protein involved in the anticoagulant mechanism [4].

Hereditary thrombophilia represents one of the risk factors among reproductive disorders. Thrombophilia expresses an important trend that finally leads to thrombosis [5]. The coagulation system may deviate to a thrombotic condition, which may be characterized as a thromboembolic pathology. This deviation is generated due to various factors, mainly because of the coagulation factors and their synergy, but likewise, because of their connection with blood components and other cells [5]. The possibility of getting diagnosed with thrombosis enhances with age, as a result of various factors, pregnancy being one of them. Venous thromboembolism (VTE) is known as a pathology expressed by multiple factors, being the ultimate clinical clue of genetic or acquired ones. A patient that may develop thrombosis should be tested for thrombophilia, so a woman with a known pro-thrombotic condition has to be evaluated as such [6]. Bates et al. confirmed in one of their latest studies that 1.2 in every 1000 deliveries are complicated by VTE. The necessity to consider both foetal and mother well-being makes diagnosis, prevention, and treatment of pregnancy associated VTE particularly problematic. Current guidelines [6] deal with these difficult challenges. Thrombophilia testing is frequently requested in clinical practice by asymptomatic persons with a family history of VTE. The presence of thrombophilia is not predicted by having a family history of VTE, but women who plan to become pregnant may benefit from thrombophilia testing [7].

## 2. Aetiology of Thrombophilia

**Figure 1:**
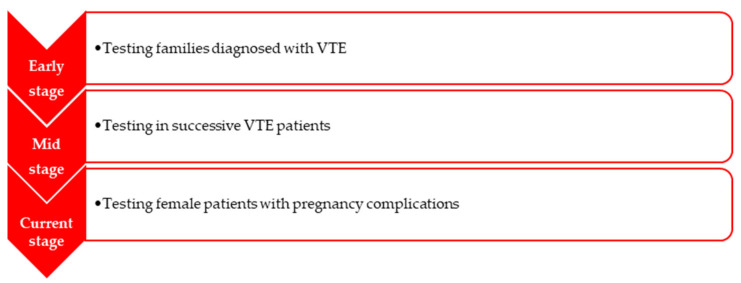
Progression of hereditary thrombophilia testing (information taken from [5]).

The phrase “thrombophilia” was first mentioned in 1937, when Nygaard and Brown characterized unexpected occlusion of large arteries, sometimes with associated VTE, but the familial habit of thromboembolic pathology was largely analysed by Jordan and Nandorff in 1956 [5]. The research of hereditary thrombophilia began by studying families with a known history of VTE [5]. Back in the 1900s, hereditary thrombophilia was considered to be a rare genetic disorder. Specific tests for hereditary thrombophilia developed throughout time [5]. The chance of a thrombotic episode to occur increases in the presence of several risk factors like aging, immobilization, prolonged orthostatism, obesity, diet, smoking, elevated oestrogen levels, birth control intakes (increases the chances of a VTE up to three times), and last but not least, pregnancy and the postpartum period [4,5]. The relation made among VTE and inherited thrombophilia was proved after performing case–control studies (Figure 1) [5]. It was estimated that 50% of the thrombotic episodes may happen all of a sudden, 30% are related to pregnancy and 20% are linked with surgery [5].

Friederich et al. confirmed in their research that if a venous thromboembolic episode happens during pregnancy or within three months of childbirth, it is pregnancy related [8]. The incidence of VTE in pregnancy increases up to five times, with a rate of 0.76-1.72 out of 1000 pregnancies, but most thrombotic events occur mostly in the puerperal period [9].

When assessing the optimal prophylaxis, a patient diagnosed with thrombophilia should be evaluated for the majority of risk factors, known as triggers for first or recurrent thrombosis. Along these lines, when concluding the thrombotic profile of the patients, genetic risk factors should be considered as well as acquired ones, considering that obesity, trauma, acute medical conditions, surgery or malignancies are defined by increased levels of procoagulant factors. In contrast, pregnancy is associated with decreased anticoagulant factors levels [1].

Knowledge about the aetiology of VTE is improving as time goes by, but screening for hereditary thrombophilia is most of the times not convenient, even though it should be noted that pregnant females have an undoubtedly elevated risk of developing VTE, especially if they have affirmative family antecedents for VTE [5].

### Particularities of the Hemostatic System in Pregnancy

The chances of bleeding and thromboembolic events are elevated during pregnancy. The utero-placental entity is unique, and its most significant function is to maintain contact between the maternal and foetal circulations, which is a necessity for foetal survival, so the circulation must be developed and sustained [10].

In humans, foetal haemoglobin (HbF) represents almost 2% of the total haemoglobin (Hb) in adults [10]. It is assumed that the physiological function of HbF enables the transition of oxygen from maternal to foetal blood. The interchange of gases such as oxygen and carbon dioxide as well as nutrients must be allowed at the boundary between the maternal and foetal circulations, so, since bleeding and thrombotic activities may impair this critical feature, the rehabilitation of the utero-placental circulation requires multiple systems [10,11]. Coagulopathies in pregnancy, such as deep venous thrombosis (DVT) and repeated pregnancy losses (RPL), may contribute to obstetric emergencies, but these events might be obviated if women would be carefully observed for warning signs that could suggest the need for testing to detect these undesired incidents [12]. Coagulopathies could be predicted by basic laboratory studies, such as a global coagulation panel like complete blood count (CBC), prothrombin time (PT), partial thromboplastin time (PTT) and plasma fibrinogen. The placenta is a highly advanced pregnancy organ that facilitates natural foetal growth and development [13]. Changes in the growth and function of placentas’ have dramatic implications on the foetus and its capacity to deal with the intrauterine habitat. In the existence of genetic thrombophilic mutations, considering the number of studies supporting or not the link between hereditary thrombophilia and pregnancy complications, it is impossible to determine the precise risk figures for serious adverse effects [11]. Pregnancy is a prothrombotic condition, and prothrombotic events are obvious as gestation advances. The development of sufficient placental circulation is needed for a healthy pregnancy, and hereditary thrombophilia can be a risk factor for placenta-mediated pregnancy adverse effects [12]. Gestation induces a 2 to 3-fold rise in fibrinogen concentrations, as well as a 20% to 1000% raise in factors VII, VIII, IX, X, and XII, all of which peak at delivery [14]. During normal pregnancy, blood coagulation factor XIII, XII, X, VIII, von Willebrand factor, ristocetin cofactor, factor VII, and fibrinogen increase dramatically, and the most pronounced improvements are observed in the third trimester [10]. These modifications guard against potentially lethal haemorrhage during pregnancy and through the third stage of gestation, but they also raise the likelihood of maternal thromboembolism [10]. In 1000 patients, the incidence VTE is estimated to be between 0.5 and 2.2. It is five times higher among pregnant women, with the highest risk of thrombosis registered between week 6 and 12 after childbirth [15]. The increased levels of oestrogen and progesterone during gestation lead to a hypercoagulable condition, resulting in an upregulation of the clotting factors and a reduction in the levels of anticoagulants [15].

Inherited thrombophilia is assumed to be present in up to 50% of venous thrombotic events that occur during gestation and puerperium, and is well-known that thrombosis during pregnancy, as well as underlying thrombophilia, may have severe consequences for both the mother and the foetus [15,16]. Biological hypercoagulability is a term used to explain variations in blood coagulation and fibrinolysis which have a thrombotic aspect during gestation, and increased fibrin turnover is a result of hypercoagulation. This is demonstrated by increased concentrations of D-Dimers (D-D), the most susceptible marker of secondary fibrinolytic activation [17].

## 3. Classification of Thrombophilias

Thrombophilia is divided in two main groups—inherited and acquired.

### 3.1. Hereditary Thrombophilia

The inheritable thrombophilic states [18] may be subdivided in two parts: common, respectively, major inherited thrombophilias [1]. Additionally, they may be classified into two categories: in the first one, inhibitors of coagulation are decreased—deficiency of coagulation inhibitor factor such as antithrombin III (AT III), protein C and protein S deficiency; in the second category, coagulation factors are elevated—activated Protein C resistance (APC), Factor V Leiden, prothrombin gene mutation, increased levels of VIII, IX, XI factors, and dysfibrinogenemias. The first category is more predisposed to thrombosis than the second one, which is more likely to be associated with the first event of thrombosis [18].

Therefore, regarding inherited thrombophilia, the most common causes are the prothrombin gene mutation G20210A and factor Leiden, concluding up to 70% of the diagnosed inherited forms of thrombophilia. The less common but most severe triggers are the defects of protein C and S and of antithrombin III [1,4,5,18].

### 3.2. Acquired Thrombophilia

Besides inherited thrombophilia, hypercoagulable conditions may also be generated by acquired pathologies of the haemostasis. Acquired states can sustain a prothrombotic condition due to increased procoagulant factors and decreased anticoagulants besides other modifications of the haemostasis [1]. The major acquired pathologies linked to thrombophilia are the following: hyperhomocysteinemia, antiphospholipid antibody syndrome (APS), elevated levels of procoagulant factors and reduced levels of anticoagulants. Normally, these levels are balanced naturally, but they can be modified due to acquired factors or because of aging [1,4]. APS is an immune mediated disorder, defined by obstetrical or thrombotic circumstances [1]. An obstetrical APS is represented by preeclampsia (PE), recurrent miscarriages, foetal growth restriction (FGR), first trimester abortion (FTA), mid-trimester abortion (MTA), placental abruption (PA) or intrauterine death [1,3], yet a thrombotic APS is defined by venous or arterial thrombosis [1]. Antiphospholipids antibodies [aPL] (primarily anticardiolipin) are responsible for setting the APS, together with the detection of lupus anticoagulant [1,3]. The diagnosis of this immune mediated disorder is confirmed if at least one of the clinical manifestations specified earlier is proven and if aPL are described in more than two occasions.

Regarding the risk for thrombosis, it may be subdivided into high risk, moderate and low risk thrombophilia; high risk thrombophilia includes antithrombin III, protein C and protein S deficiency, moderate risk thrombophilia consists of factor V Leiden, prothrombin gene mutation and factor VIII, and last but not least, low risk thrombophilia covers for factor IX, factor XI and hyperhomocysteinemia [19].

### 3.3. Grading the Risk

#### 3.3.1. High Risk Thrombophilia

##### Antithrombin III Deficiency (AT III)

AT III is one of the essential plasmatic inhibitors for the activated coagulation factors, immediate aim being for sure thrombin [20]. Deficiency of AT III is established to be a risk factor for venous thrombosis, considering that acquired deficiencies are more common, but it may be fixed by administrating the proper anticoagulation regimen [20]. When heterozygous mutations are detected in the AT gene, this may be followed by AT defects, which are defined by decreased inhibition of factor Xa [1]. Measuring the level of AT III should be performed before administrating the anticoagulation with heparins or with low molecular weight heparins. It is established to increase the risk of thrombosis up to 50%.

##### Protein C and S Deficiencies

Protein C and S deficiencies, rare pathologies are described by a reduction in the activity of protein C, respectively S, [21,22]; protein C deficiency was identified by Mammen et al. in 1960, yet Stenflo et al. named it the way we know it today. It is known to increase the risk of thrombosis up to 20%, unlike protein S, which increases this risk up to 10%. Thus, hereditary deficiency of both protein C and S leads to an elevated thrombin production and predilection to thrombosis [1].

#### 3.3.2. Moderate Risk Thrombophilia

##### Activated Protein C Resistance and Factor V Leiden

Activated Protein C Resistance was described for the first time in the early 1990s by Dahlback and Hildebrand [18]; they discovered an error regarding the anticoagulant response while activating protein C (APC). APC resistance was demonstrated to be inherited and linked with hereditary thrombophilia [23]. Factor V Leiden, a hereditary defect of haemostasis, and also a low risk factor for thrombosis, linked with the possibility of developing first and recurrent venous thromboembolism, was included in various screening programs. It is also associated with thrombosis, particularly in women known with other risks factors, such as pregnancy, older age, oral contraceptive intakes, hyperhomocysteinemia, and deficiencies of protein C and protein S [18,24].

##### Prothrombin Gene Mutation (Factor II)

Prothrombin, or factor II, precursor of the thrombin [25], was first mentioned in 1996 by Poort et al. [18]; prothrombin G20210A mutation is known for the changing of the guanine nucleotide (G) with the adenine nucleotide (A) in the 20210 position, and it affects up to 6% of the population, increasing eventually the risk of thrombosis. Even though its heterozygous posture is known as a low risk factor for developing associated VTE complications, this risk is increased during pregnancy [25]. Nevertheless, both oral contraceptives intakes and pregnancy eventually develop an increased prothrombin activity [25].

##### Factor VIII

Patients with records of VTE have presented elevated levels of factors VIII, IX and XI [18], although it is uncertain how increased levels of coagulation factors interfere with the risk of thrombosis, but once the level of factor VIII increases, so does the risk of thrombosis (up to 10%).

#### 3.3.3. Low Risk Thrombophilia

##### Hyperhomocysteinemia

The demethylation of methionine forms the amino-acid-homocysteine [1]. Homocysteine is a commonly developing amino-acid [26]. Increased levels of homocysteine, called hyperhomocysteinemia may result from all sorts of hereditary factors or from a diet poor in folic acid and B vitamins. Elevated levels in pregnancy may end up with recurrent pregnancy loss, but the presence of methylenetetrahydrofolate reductase gene (MTHFR) in the absence of elevated levels of homocysteine is not linked with a significant risk of thrombosis [1].

## 4. Thrombophilias and Complications in Pregnancy

Pregnancy is a state described as carrying a foetus within the female body that usually ends through miscarriage or delivery [27]. Hereditary and acquired thrombophilias are responsible for more than 50% of the thrombotic events diagnosed during pregnancy and the postnatal period [28]. Along with birth and the postnatal period, haemostatic problems may occur, which involve important complications regarding morbidity and mortality for the mother and foetus as well [29]. Because pregnancy and the postnatal period are well known risk factors for thrombosis, it is established that pregnant women may develop venous thromboembolism in 50% of the cases. On the other hand, any pregnant woman deals with physiological adjustments during pregnancy that end up interfering with biochemical parameters. These adjustments lead to a hypercoagulable condition, so the risk of venous thromboembolism affects 1 in 1600 births [30].

Haemostasis is a vital equilibrium that includes the natural anticoagulation system, fibrinolysis and procoagulants [31]. Normal pregnancy is linked with significant changes in the haemostasis [31], so the procoagulants outcome end up being predominant. Serious pregnancy complications—preeclampsia (PE), recurrent miscarriages, fetal growth restriction (FGR), first trimester abortion (FTA), mid-trimester abortion (MTA), placental abruption (PA) or intrauterine death [3,32]—increase both maternal and foetal morbidity and mortality [28]. Adequate uterine blood flow and normal placental evolution contribute to a typical pregnancy outcome, which is related with significant changes in the coagulation and anticoagulation process, and there is noticed an important boost towards the coagulation factors—fibrinogen, prothrombin, VII, VII, X and XII [28].

On the other hand, in pregnancy, anticoagulant levels might increase lightly (tissue factor pathway inhibitor -TF- the primary initiator of coagulation) [33]; they may also remain stable (antithrombin III and protein C) or they may diminish unquestionably (protein S).

Thrombophilia is known as a pathogenic factor for serious pregnancy complications. Modifications of the procoagulants factors—mutant genes with great prevalence able to increase the risk in developing thrombosis have been studied [28,30]: homozygous or heterozygous mutation in the methylenetetrahydrofolate reductase gene (MTHFR) in the C677T and A1298C positions, the homozygous or heterozygous mutation of the factor V Leiden (FVL) gene in the G1691A position, homozygous or heterozygous mutation of the prothrombin gene in the G20210A position (factor II) or the polymorphism of plasminogen activator inhibitor-1 (PAI-1) 4G/4G mutations [28,30,34].

A favourable result of a pregnancy is related to normal placental formation [34]. Coagulation factor XIII has an important impact regarding its formation, since it is implied in cross-linking fibrin and it is known to be affecting the fibrinolysis. For factor XIII, a frequent polymorphism is connected with an early cross-link and a diminished perceptivity to fibrinolysis. Therefore, factor XIII Val34Leu polymorphism may destroy fibrinolysis, and might increase the general risk for RPL [34]. An equivalence between thrombosis, pregnancy complications and thrombophilia persists to be studied [30].

### 4.1. Screening Options

Screening for thrombophilia does not interfere with the proper management practiced in pregnancy, recurrent pregnancy losses or infertility issues. A study conducted by Ashraf in 2019 highlighted findings from 1995 to 2017 regarding the importance of genetic thrombophilia testing, in patients that were susceptible of having hypercoagulable conditions [35]. Thrombophilia screening is questionable [36]. Nowadays, laboratories performing various sets of tests regarding the diagnosis of thrombophilia are developing quickly [37]. Screening for hereditary thrombophilia was first mentioned in 1965, when a family was diagnosed with a deficiency of the serine protease inhibitor antithrombin. Later, a few irregularities have been linked with hereditary thrombophilia, and a vast set of tests have been implemented by laboratories in order to identify patients with these irregularities. Testing for hereditary thrombophilia implies a wide variety of coagulation and genetic tests [38], although testing is costly, and interpretations require clinical competence. Specific tests for thrombophilia should not be performed during a thrombotic episode, because the results may be influenced by a few factors, and yet the existence of inherited thrombophilia does not interfere with the primary management of VTE. Screening for thrombophilia involves an ample set of tests, that may include the following parameters: prothrombin G20210A, factor V Leiden (FVL), factor V HR2, factor XIII V34L, plasminogen activator inhibitor-1 4G/5G (PAI-1), methylene tetrahydrofolate reductase (MTHFR) C677T, MTHFR A1298C, β-fibrinogen-455 G>A, apolipoprotein E (Apo E), angiotensin-converting enzyme I/D, apolipoprotein B R3500Q [39]; measurements of protein C, protein S, antithrombin III are also performed, as well as analysis of homocysteine levels [38], and last but not least, tests to distinguish values of lupus anticoagulant, anticardiolipin antibodies and anti-beta 2 glicoprotein-I antibodies in order to confirm or infirm the presence of antiphospolipid syndrome (APS) [37,40]. It is essential to be aware of the drugs taken at the time of testing—for instance, while administrating low molecular weight heparin (LMWH), antithrombin levels may decrease [38]. It is well known that screening for acquired conditions of thrombophilia should be taken into consideration in all situations of thrombosis, although testing for hereditary conditions is improbable to be always useful [41].

Therefore, when should specific tests be performed and who should be tested? In most of the cases, where VTE is suspected and where trigger factors are obvious. Determining proper conditions in order to initiate screening may be useful for the patient as soon as a suitable treatment is prescribed.

Thrombophilic states should be studied in order to be able to take precautionary measures such as: improved prophylaxis in case of pregnancy, avoidance of combined oral contraceptive pill intake (OCP) or of hormonal therapy [41]. Inherited thrombophilia may be associated with VTE in pregnancy, and it may lead to complications such as recurrent pregnancy loss. Based on consensus expert opinion, a manuscript published in 2016 by the Anticoagulation Forum provides practical clinical guidelines on the prevention and specific treatment of obstetric-associated VTE [42].

Screening for thrombophilia in pregnancy is tempting and is widely used, because it raises the occasion of prescribing antiplatelet drugs and anticoagulant therapy in order to reduce the risks of thrombotic events. Thrombophilia, a disorder that finally leads to thrombosis, is linked with an important risk of VTE, especially in women who are prescribed OCP, hormonal therapy or who are pregnant. A few analysis performed over the years have tried to set up the cost effectiveness of testing for thrombophilia. In a study conducted by Wu et al. [43], it was established that performing routine screening for thrombophilia in women before pregnancy it is not profitable, economically speaking [41]. This cost effectiveness study was carried out through UK National Health Services, in order to establish the cost effectiveness of common and selective screening, regarding personal or/and a family history of VTE in comparison with no testing for thrombophilia. The unfavourable complications were linked with screening and no screening in the assumed groups, so four screening theories were tested, as follows: screening 10.000 women in each theory—prior OCP intake, at week 6 of pregnancy, prior prescribing hormonal therapy and last but not least, prior surgeries [43]. The women from the pregnancy group, who were tested positive for thrombophilia, in order to prevent RPL and VTE, would be prescribed prophylaxis drugs. In the common testing, all the patients would be screened; still, in the selective testing, only the patients known with a previous VTE or a family history of thrombosis would be screened. Therefore, in an assumed model of 10,000 patients, where the routine testing has not been performed, unfavourable complications would be encountered in 7 women on OCP, 2921 pregnant ones, 104 patients prescribed hormonal therapy and 1265 women subject to surgeries [43]. Regardless, thrombophilia screening still remains questionable [36].

### 4.2. Clinical Evaluation

A clinical evaluation for thrombophilia should be performed for all of the patients who had experienced an episode of VTE [44], or for women with a known personal history of preeclampsia, recurrent miscarriages, foetal growth restriction, first trimester abortion, mid-trimester abortion, placental abruption or intrauterine deaths [1,3]. The evaluation starts with an accurate family and personal history for thrombosis, past clinical antecedents, associated pathologies and interpretation of the existing risk factors. An entire physical examination is recommended, paying increased concentration to skin, lymphatic, peripheral arterial and venous, cardiorespiratory, abdominal, urinary and neurological system.

VTE may be separated in idiopathic–unprovoked and secondary-provoked. Provoked VTE is characterized by transitory–reversible, minor and major risk factors or by irreversible–persistent ones; thrombophilic conditions should be evaluated in order to improve prophylaxis in case of the existence of the following risk factors—non-provoking ones—age, sex, combined oral contraceptive pill intake (OCP) or of hormonal therapy, and of provoking ones—pregnancy, long-distance-travel, trauma, surgery, immobilization or cancer [42,44]. Distinction between forms of VTE influences the treatment judgment.

One in 1000 pregnancies is known with associated VTE, and the risk of thrombosis is higher in the postpartum period [45,46]. The majority of the symptoms that may claim deep venous thrombosis (DVT) like dyspnea, tachycardia, groin discomfort, unilateral leg pain and swelling are known as physiological transformations in pregnancy, and eventually most of the women presenting these changes will not develop DVT, but the risk of pregnancy-associated-VTE is increased up to ten times in this category, unlike the non-pregnant same age control-lot [45]. Women with a history of recurrent miscarriages are investigated for thrombophilia in order to set the proper pregnancy-management [45].

### 4.3. Methodology and Patient Management

Inherited thrombophilias are associated with early pregnancy loss, taking into account an important risk of VTE. This state continues to be a valuable cause of maternal morbidity and mortality [47]. Pregnancy is known to have a five-fold increase in developing VTE, this risk being multiplied up to twenty times during the postpartum period [48]. Due to probable maternal and foetal complications, a proper therapeutic strategy during pregnancy is quite demanding. Various therapeutic methods have been taken into consideration regarding the management of pregnant women diagnosed with hereditary thrombophilia, such as: anticoagulants, antiplatelets drugs and vitamins [6,42,49]. When choosing an ideal treatment for a pregnant woman with inherited thrombophilia, additional risks must be deliberated, regarding its efficacy and safety. Evidence and guidance are followed in order to establish clinical decisions regarding duration of thrombolytics and anticoagulation in pregnant women diagnosed with hereditary thrombophilia. The novel guidelines of American Society of Hematology for the management of venous thromboembolism in the context of pregnancy are improved from the ones followed in 2012 and in 2016 [6,42,50].

#### 4.3.1. Anticoagulants during Pregnancy

When inherited thrombophilia is confirmed, antepartum prophylaxis should be initiated immediately, since the risk of VTE seems to develop early in pregnancy [49]. The administration of anticoagulants during pregnancy is challenging, due to possible associated complications [47]. Likely side effects of maternal anticoagulant treatment may include teratogenicity, bleeding, and recurrent pregnancy loss [47]. The anticoagulants of choice are low-molecular-weight heparin (LMWH) and unfractionated heparin (UFH), owing the ability of not crossing the placenta, being safe for the foetus [47]. Vitamin K antagonists, warfarin for instance, crosses the placenta, being disclosed as potential causer for the enumerated complications—for example, it may lead to the hypoplasia of the nasal bone, if it is administrated between weeks 6 and 12 of pregnancy, or it may cause anomalies of the central nervous system in any trimester [47]. In a study conducted in vitro in 2002 [49,51], there was no trace of transplacental transfer of fondaparinux, which is a synthetic pentasaccharide, an antithrombotic agent that selectively inhibit coagulation factor Xa. Even though it has been described being a safe agent, it may cause foetal teratogenicity, so its usage is mainly restrictive. LMWH known for their elevated bioavailability, have shorter polysaccharide chains and lower molecular weights unlike UFH, with no need in performing regular coagulation tests [52,53]. LMWH can be self-administrated, subcutaneously once a day. If possible, checking out anti Xa levels and testing D-Dimers should be performed monthly during a pregnancy, in order to prevent secondary complications [53,54]. As all heparins, LMWH dosing is based on a patients’ weight, but administrating it in accordance to anti Xa levels remains disputed [47]. The optimal therapeutic dose for prophylaxis is not clear, and it has not been demonstrated if dose adaptation in order to maintain clear-cut anti Xa levels increases prophylactic efficacy or safety [47]. On the other hand, UFH may be linked with thrombocytopenia, osteoporosis and maternal bleeding, so LMWH are preferred for a long-term prophylaxis [53]. When extended anticoagulation is needed during pregnancy, maternal complications may occur as well. All anticoagulants are likely to cause bleeding, allergic reactions or pain at the injection’s sites—but this should not lead to the interruption of the treatment [52]. While hereditary thrombophilias are mostly linked with an inclination to VTE, acquired forms are associated with both venous and arterial events. APS represents an acquired form of thrombophilia, defined by the presence of antiphospholipid antibodies (aPL), clinically characterized by arterial or venous thrombosis. This is also associated with obstetric complications, the diagnosis being based on the Sydney criteria: one clinical criteria (pregnancy morbidity or arterious or venous thrombosis) and one laboratory criteria (high value of lupus anticoagulant, of anticardiolipin antibodies IgM/IgG or of anti-beta 2 glicoprotein-I antibodies IgM/IgG) [40,49,54,55]. Although antiphospolipid syndrome (APS) was first identified as an autoimmune thrombophilia, we now know that additional mechanisms besides coagulation-mediated thrombosis play a role in various clinical presentations; for example, complement activation may play a role in placental damage, which can result in fetal loss [56]. Even though anticoagulants and thrombolytics are the most administrated drugs in order to prevent pregnancy-related complications in women diagnosed with hereditary thrombophilia (Table 1) [54], at the time of delivery, the risk of haemorrhage may occur in anticoagulated women, but it can be reduced with careful preparation (Table 2) [49].

Anticoagulants should be started postpartum as soon as proper haemostasis is established [54]. LMWH can be initiated 12–24 h postpartum in deliveries without associated complications. If warfarin is preferred to be used post-delivery, it may be initiated exactly like LMWH. Not only warfarin, but also LMWH is safe for breastfeeding patients. Postpartum prophylaxis is recommended for nearly six weeks, with prophylactic doses of LMWH or UFH, with D-Dimers tested regularly [49,54]. Clinical trials evaluating oral anticoagulants such as direct thrombin and anti Xa inhibitors, as well as apixaban agents, excluded pregnant women from taking part in them, taking into account that potential foetal or maternal complications are not known, so the use of this kind of anticoagulants should be avoided [55].

#### 4.3.2. Thrombolytics during Pregnancy

Antiplatelets drugs cross the placenta, and a few studies performed on animals showed that thrombolytics may boost the risk of congenital abnormalities, but the information obtained from studies performed on human subjects is contradictory [55,57]. However, aspirin (acetylsalicylic acid) may be administrated in pregnancy for explicit indications, considering that in the course of the second and of the third trimester of pregnancy, it has not been associated with an increased risk of pregnancy loss. A meta-analysis conducted by Kozer [57,58,59] found no significant risks regarding congenital abnormalities that were linked with aspirin intake, among exposed infants, but it may lead to gastroschisis if taken during the first trimester—five case–control reports confirmed this risk [60]. When questioning if antiplatelets are appropriate to be prescribed in high risk pregnancies, probable risks should be considered as well [57].

### 4.4. Prevention

#### Prevention for Adverse Pregnancy-Related Complications

Women who accomplish the APS syndrome criteria, and are also known with three or more recurrent pregnancy losses, are advised to take antepartum prophylactic or intermediate dose of UFH, or prophylactic doses of LMWH associated with aspirin 75 mg up to 100 mg daily [55]. Nonetheless, the results obtained from one multicenter randomized trial, which was based on the association of LMWH with low-dose aspirin, initiated before the 12th week of pregnancy, showed a reduction in early pregnancy losses in women diagnosed with hereditary thrombophilia. Taken together, women known to have hereditary thrombophilia, treated with a combination of prophylactic doses of LMWH and aspirin, initiated before the second trimester of gestation, have had a reduced risk of miscarriage [58].

Treatment choices are questionable. A conventional antithrombotic treatment option for primary and secondary prophylaxis regarding thrombotic events during pregnancy includes anticoagulation LMWHs. Pregnancy raises the risk of VTE, so pharmacological prophylaxis is usually prescribed for women who have had a previous episode of VTE during pregnancy [59]. Heparin and its subsidiaries may strive a helpful effect by limiting gestational vascular complications [60]. Regarding thromboprophylaxis, it has been proven that LMWHs and low dose aspirin (ASA) are really effective in pregnancy, with excellent live birth rates.

### 4.5. Prognostic Outcomes

Predicting the risk of VTE continues to be challenging and although various efforts have been accomplished in order to establish predictive-biomarkers, only D-dimers remain the most used one [61]. Preeclampsia (PE) is one of the most frequent pregnancy complications that leads to maternal morbidity and mortality globally, and it may be triggered by genetic conditions such as thrombophilia [3,62].

## 5. Micro RNAs in Pregnancy

The study of micro RNAs (miRNAs) in thrombophilia seeks for a lot of debate. MicroRNAs are small, single-stranded endogenous RNAs that regulate the expression of different target genes post-transcriptionally [63]. The first miRNA, lin-4, was discovered in 1993 as a 22-nucleotide non-coding RNA that controlled the timing of post embryonic development in Caenorhabditis elegans by repressing lin-14 protein expression, and let-7, the second miRNA discovered, was analysed in nematodes. Nowadays, there are known more than one thousand human miRNAs, each likely regulating hundreds of target genes, and they act as valuable gene regulators to regulate different physiological events, including placental development [63].

During pregnancy, the placenta is a transient organ, acting as the junction between maternal and foetal setting. Expanding evidence indicates that miRNAs are important regulators of placental growth [64].

Research on prognosis is significant. More women are now living with hereditary thrombophilia, and nowadays prognosis research seeks to understand and enhance future outcomes, and it provides critical evidence to translate findings from laboratory to human, and from clinical research to clinical practice [65].

In a study conducted in 2020 [66], there were families who had experiences with an idiopathic thrombosis episode—except those with antithrombin deficiency or who have a mutation in factor V Leiden, that volunteered to participate. Patients ceased treatment with oral anticoagulants and antiplatelet medications (slightly 15 days before extraction) as well as heparin medication prior to collection of blood samples (partly 24 h before). This study that analyses the connection between miRNA production and VTE revealed 16 miRNAs of interest that were identified. Therefore, genetic and epigenetic studies are needed to find biomarkers of thrombosis and to identify their clinical applications in the discovery phase, and the internal validation phase showed four of those to be differentially expressed in patients with VTE: hsa-miR-126-3p, hsa-miR-885-5p, hsa-miR-194-5p, and hsamiR-192-5p [66].

Not only did the four validated miRNAs display a big association with VTE, but they are also possible predictors of this pathology, therefore, four differentially expressed plasma miRNAs are described in VTE [66].

It has been estimated that the rate of spontaneous pregnancy loss is 30%; the human placenta has been shown to contain a significant number of miRNAs that are implicated in its growth [67]; as in more, during the peri-implantation cycle, miRNAs control uterine gene expression associated with inflammatory responses and participate in maternal–foetal immune tolerance [67]. The correlation of aberrant miRNA expression with different human diseases linked to reproductive conditions has been seen in various studies [62].

It is important to establish whether genetic polymorphisms display a correlation with idiopathic recurring pregnancy loss in miRNA machinery genes.

High quality research is important in order to define pathways by which polymorphisms of miRNA machinery genes influence the production of RPL, considering these wide potential uses of prognostic factors [67].

The majority of studies on birth cohorts utilizing miRNAs as biomarkers in pregnancy have focused on determining the link between environmental contaminants and alterations in the miRNA-ome in placental samples [68].

## 6. Conclusions

The main objective of the study was to quantitatively assess the literature data associated with the management of pregnant women with thrombophilia. An essential element would be the rectification of the notion of thrombophilia and an appropriate preventive activity instituted before pregnancy. Since in most cases the pregnant woman is exposed to coagulation disorders occurring during pregnancy, an equally important element is to adapt the general management of the pregnant woman to the particularities generated by thrombophilia. Thus, the following directions of approach to the management of pregnant women with thrombophilia are outlined:

- In screening, the degree of genetic damage that manifests as thrombotic risk is assessed. Depending on this, treatment with anticoagulant and antiaggregant drugs is tailor-made for each patient.
- Following the high degree of risk amplified by the evolution of pregnancy, the frequency of patient monitoring will be increased to avoid any thrombotic event that could endanger pregnancy.
- Postpartum, antithrombotic prevention will be maintained, therapy being adapted to the degree of risk associated with the severity of thrombophilia.

A future direction in the management of pregnant women with thrombophilia will include mandatory microRNA profiling, which will be a useful tool in both diagnosis and monitoring and prognosis.

## Figures and Tables

**Table 1:** Preferred prophylaxis in order to prevent pregnancy-related complications in women diagnosed with inherited thrombophilia—summary of recommendations (American College of Chest Physicians).

Prevention of Pregnancy-Related Complications	Recommendations
No previous VTE HIGH RISK: homozygosity for the mutation of factor V Leiden or the presence of prothrombin gene mutation	
✓ Absence of a family history of VTE:	➢ antepartum clinical surveillance ➢ post-delivery prophylaxis up to 6 weeks
✓ With a positive family history of VTE:	➢ antepartum prophylaxis
	➢ post-delivery prophylaxis
LOW RISK: other forms of thrombophilias	
✓ Absence of a family history of VTE:	➢ antepartum and post-delivery clinical surveillance
✓ With a positive family history of VTE:	➢ antepartum clinical surveillance ➢ post-delivery prophylaxis up to 6 weeks
Previous VTE MODERATE AND HIGH RISK:	➢ antepartum prophylaxis
One or multiple episodes of VTE:	➢ post-delivery prophylaxis up to 6 weeks
LOW RISK: One episode of VTE, linked with a temporary risk factor, that is not pregnancy-related:	➢ antepartum clinical surveillance

Information taken from [25,54,55].

**Table 2:** Management at the time of labour.

Administrated Agents— Should Be Interrupted 12–24 h Prior to Elective Induction of Delivery	Recommendations If Unplanned Delivery Happens, Neuroaxial Anaesthesia Should Not Be Performed
UFH	➢ checking activated partial thromboplastin time and considering administrating protamine sulphate in case it is prolonged to reduce the risk of bleeding.
LMWH information taken from [49]	➢ checking out anti Xa levels and testing D-Dimers; ➢ in case of bleeding, protamine sulfate may offer limited neutralization; ➢ major bleeding—recombinant activated factor VII concentrate in case there is lack of response to classic therapy;

## Data Availability

Not applicable.
